# Rethinking Integrated Care: A Systematic Hermeneutic Review of the Literature on Integrated Care Strategies and Concepts

**DOI:** 10.1111/1468-0009.12459

**Published:** 2020-05-20

**Authors:** GEMMA HUGHES, SARA E. SHAW, TRISHA GREENHALGH

**Affiliations:** ^1^ Nuffield Department of Primary Care Health Sciences University of Oxford

**Keywords:** integrated care, health systems, chronic care, hermeneutic review

## Abstract

**Policy Points**

- Integrated care is best understood as an emergent set of practices intrinsically shaped by contextual factors, and not as a single intervention to achieve predetermined outcomes.
- Policies to integrate care that facilitate person‐centered, relationship‐based care can potentially contribute to (but not determine) improved patient experiences.
- There can be an association between improved patient experiences and system benefits, but these outcomes of integrated care are of different orders and do not necessarily align.
- Policymakers should critically evaluate integrated care programs to identify and manage conflicts and tensions between a program's aims and the context in which it is being introduced.

**Context:**

Integrated care is a broad concept, used to describe a connected set of clinical, organizational, and policy changes aimed at improving service efficiency, patient experience, and outcomes. Despite examples of successful integrated care systems, evidence for consistent and reproducible benefits remains elusive. We sought to inform policy and practice by conducting a systematic hermeneutic review of literature covering integrated care strategies and concepts.

**Methods:**

We used an emergent search strategy to identify 71 sources that considered what integrated care means and/or tested models of integrated care. Our analysis entailed (1) comparison of strategies and concepts of integrated care, (2) tracing common story lines across multiple sources, (3) developing a taxonomy of literature, and (4) generating a novel interpretation of the heterogeneous strategies and concepts of integrated care.

**Findings:**

We identified four perspectives on integrated care: patients’ perspectives, organizational strategies and policies, conceptual models, and theoretical and critical analysis. We subdivided the strategies into four framings of how integrated care manifests and is understood to effect change. Common across empirical and conceptual work was a concern with unity in the face of fragmentation as well as the development and application of similar methods to achieve this unity. However, integrated care programs did not necessarily lead to the changes intended in experiences and outcomes. We attribute this gap between expectations and results, in part, to significant misalignment between the aspiration for unity underpinning conceptual models on the one hand and the multiplicity of practical application of strategies to integrate care on the other.

**Conclusions:**

Those looking for universal answers to narrow questions about whether integrated care “works” are likely to remain disappointed. Models of integrated care need to be valued for their heuristic rather than predictive powers, and integration understood as emerging from particular as well as common contexts.

Health care systems around the world are being redesigned. An increase in aging populations and associated multimorbidity in high‐income countries requires a shift from episodic treatment of acute illness toward greater continuity of care for chronic conditions. Greater alignment of health and social care is also required to prevent unnecessary hospital admissions and to avoid the dangers of duplicated, fragmented, and uncoordinated care.

A guiding principle for system redesign is integrated care. *Integrated care* refers to both the methods that might be used to organize, fund, and deliver health and related services and the interrelated goals of better outcomes, experiences, and use of resources. Integrated care has been studied in various ways—for example, as an organizational and social process,^1^ as an indicator of health system effectiveness,^2^ and for its effects, such as economic impact.^3^ The result is a heterogeneous body of literature with the term used by different authors to mean different things. Most espouse “patient‐centered” goals and values. Few dispute the principle of integration. (Who would want care that is *not* integrated?)

To date, consistent benefits from integrated care programs have proved elusive. Despite evidence of some aspects of improvement in certain settings for certain people, expectations that integrated care programs will improve outcomes and reduce health service utilization are often disappointed.

In this paper, we set out the methodology and findings of a systematic literature review aimed at deepening understanding of what integrated care is, how it is experienced, and how it is conceptualized. Contrary to much of the current literature, our findings show that integrated care is not a unified concept but is better understood as an emergent set of practices, such as multidisciplinary case management and strategic partnership working. Integrated care programs are shaped by contextual factors, such as payment systems for health services, and therefore are unlikely to reliably affect a predetermined set of outcomes. On this basis we argue that the concept of integrated care as a unified framework, and its associated strategies, requires fundamental rethinking.

## Background

Integrated care programs have been researched and evaluated to understand the extent of change and effect on a range of outcomes, from patient experience to economic impact. Demonstration projects, pilots, and managed programs of change to improve care for people who require long‐term, coordinated care (typically older people with chronic multimorbidity) have been established across high‐income countries, including Australia, Canada, Italy, the Netherlands, New Zealand, Sweden, the United Kingdom, and the United States.^4^, ^5^ Integrated care programs are also associated with longitudinal policy and system reforms—for example, in Sweden^6^ and Canada.^7^ The United Kingdom has seen a succession of programs of change (case management,^8^ older people's pilots,^9^ integrated care pilots,^10^ integrated care pioneers,^11^ and vanguards^12^) shaped by national policies to develop more integrated models of care.^13^, ^14^

The patient perspective is generally considered to be the organizing principle of integrated care, recommended to policymakers as an alternative to supply‐driven models.^15^ From the patient perspective, organizational divides between health and social care can cause duplication (eg, multiple assessments by different services) or gaps in care when transitioning from one setting to another (eg, from a hospital to a home or community). Specialization of health care treats each medical condition separately, with increased potential for fragmented, suboptimal care that fails to consider interrelated health and social care needs. Person‐centered and coordinated care is intended to address such fragmentation and to “activate” patients to engage in care planning, decision making, and self‐management.

If the principle of integrated care is the patient perspective, the object of integration should be patient care, rather than the organizations or services providing care.^16^ However, many programs of change have prioritized linkages between services and organizations to bridge traditional divides between the “cure and care” sectors in the United States,^17^ health and social care in the United Kingdom,^18^ and long‐term and hospital care in Europe.^19^ Integrated care is thought to address fragmentation of welfare and public services and thereby improve outcomes for vulnerable populations such as veterans and homeless people.^20^ Linking the health care system with wider community resources is thought to help meet the needs of chronically ill patients, as per the chronic care model (CCM),^21^ and in promoting good health.^22^

Measuring the effects of integrated care is not straightforward. Studies have found some evidence of reduced health service use^4^, ^23^, ^24^ and positive effects on quality of care,^23^, ^24^ but little evidence that integrated care can reduce direct or indirect costs^23^, ^25^ or improve cost effectiveness.^3^ Meta‐analyses and economic evaluations of integrated care programs have highlighted the inherent methodological challenges of (1) aggregating data on diverse and even contradictory outcome measures and interventions, (2) attributing causation to cumulative programs of change, and (3) distinguishing between contextual factors and interventions.^24^, ^26^, ^27^ Such methodological challenges have led some to propose an alternative framing of integrated care: as an overarching strategy rather than an intervention to be evaluated.^3^

Scholars of integrated care have analyzed the heterogeneous literature by identifying factors, principles, or heuristics (“laws”) that help explain success or failure. Approaches to the evidence on integrated care include categorizing empirical programs to identify commonalities,^28^, ^29^, ^30^ realist reviews,^31^, ^32^ and evidence synthesis.^2^, ^4^, ^33^, ^34^, ^35^ Evidence synthesis has informed development of the CCM^21^ and classifications of types of integration to develop explanatory frameworks for success.^16^, ^36^, ^37^, ^38^ Studies of the extent to which national health systems are integrated have provided generalizable principles and characteristics associated with high performance.^2^, ^39^

Empirical evidence of consistent benefits of integrated care and chronic care models remains elusive despite extensive transformation programs.^40^ Yet policy enthusiasm appears undimmed. Strategy for the English National Health Service (NHS),^14^ for example, retains integrated care (in the form of Vanguards—programs leading the way in developing new models of care) as a means for slowing the growth of hospital admissions. This policy persists despite a lack of clarity about improvement mechanisms and a history of disappointing outcomes of integrated care in the United Kingdom in terms of system and patient improvements.^8^, ^11^, ^41^, ^42^, ^43^

In sum, integrated care is an important idea that is firmly inscribed in health policy across the globe.^44^ However, the evidence, due in part to methodological challenges, fails to keep pace with policy expectations of what integrated care can achieve. Whereas much work has been undertaken to evaluate strategies and unify concepts of integrated care, less attention has been paid to the inherent diversity and tensions contained within such a broad‐ranging project. We therefore set out to make sense of the literature on integrated care by asking four questions:

- How has integrated care been defined and understood by different scholars?
- What kinds of changes have been attempted to achieve integrated care?
- What is known about patients’ perspectives on integrated care?
- What are the interpersonal, organizational, and economic features of integrated care?

Given our interest in health system concerns relating to aging populations and increasing multimorbidity, we focused our review on high‐income countries.

## Methods

With a large and heterogeneous literature on integrated care, answering the questions just described entailed identifying and synthesizing a diverse set of empirical studies and conceptual frameworks. We employed a hermeneutic approach that involves “a practical achievement through a dialogue between the reader and the text, between readers and between texts.”^45^ ^(p26)^ Guided by this interpretive and iterative approach, our research questions developed over time as we critically engaged with the literature, allowing us to develop an integrative understanding of different aspects of the phenomenon,^46^ which was particularly appropriate for a review of integrated care.^47^

As is usual for a hermeneutic approach, our review entailed repeated cycles of searching, filtering, and interpretation across wide‐ranging academic sources, policy, and gray literature. We followed (and repeated) the two interrelated cycles of hermeneutic review: search/acquisition and wider analysis/interpretation^45^ across three main phases of work: (1) initial engagement with the literature through comparison of policy, gray literature, and evidence reviews, (2) search and analysis of biomedical and social sciences framings of integrated care, and (3) synthesis of all included papers. Although we represent this process as a series of distinct steps toward a final interpretation, in practice, phases of work overlapped as our interpretation developed iteratively over a period of more thantwo years.

Our initial engagement with the literature came from a concern to understand the discrepancy between optimistic framings of evidence in policy documents and more equivocal conclusions from meta‐analyses. GH read widely across UK policy and gray literature, tracking research evidence cited and transfer of evidence from the US health system to the English NHS. Discussion among all authors concluded that integrated care was consistently framed as being required to address increasing burdens of chronic disease associated with aging and being able to offer system benefits of reducing health service cost and utilization as well as improved patient experiences. This broad framing of integrated care, combined with our interest in systemwide changes, led us to focus on programs of change, that is, planned, complex, and multilevel activities^24^ using a range of techniques^4^ to introduce new working practices between and across different organizations. We were interested in both intraorganizational changes and interorganizational linkages. Based on this initial reading of policy and evidence, we agreed to focus on programs of change, patient perspectives of care for people with multiple needs associated with aging and chronic conditions, and linkages between services and organizations.

A preliminary database search focused on (1) interventions in home/community settings for people with multiple chronic conditions associated with aging intended to affect service utilization, and (2) professionals, services, or organizations working together across boundaries to achieve a patient‐centered approach (see Appendix). Our choice of outcomes relating to service utilization necessarily limited the scope of the search but accurately reflected the aims of integrated care that we found when first engaging with the literature. Papers from this initial stage shared a broadly biomedical framing of integrated care that GH mapped and classified^45^ by tabulating the following data in an Excel spreadsheet: the main questions addressed, methods used, and rationales (lines of argument within each paper) given for how integrated care had or failed to have an effect on the population of interest. These lines of argument were, in effect, how authors made sense of their findings in relation to research questions. We then hand‐searched social science journals to extend the range of theoretical explanations of integrated care, identifying papers that brought social theory to analysis of integrated care, and incorporated these into our spreadsheet.

We used the concept of “story lines of research”^48^ to synthesize diverse studies, including reviews and primary research. These story lines were in effect the lines of argument (following Noblit and Hare's approach to synthesis^49^) that we interpreted *across* papers, as distinct from the rationale within each paper. To synthesize the literature, we took each underpinning research question as our unit of analysis, asking what was each author trying to know (through whichever methodological framework employed)? This enabled us to bring papers with different methodological framings into conversation with each other. We distinguished between 2 story lines: concepts—that is, story lines explaining what integrated care is (eg, by theorizing empirical findings)—and strategies, or story lines explaining how to “do” or accomplish integrated care. These categories were not mutually exclusive, with, for instance, a distinct body of literature bringing together concepts and strategies to address concerns of measurement.^50^, ^51^, ^52^, ^53^, ^54^ The distinction between concepts and strategies allowed us to compare how integrated care has been conceptualized and implemented to find different explanations of how integrated care can manifest and affect change (eg, Embuldeniya, Kirst, Walker, and Wodchis^1^) or that explained findings as to why change had not been detected (eg, Huntley et al.^55^).

We critically appraised lines of argument and story lines to make sense of how different studies addressed their underpinning questions. We filtered out papers that failed to convince us of their line of argument in light of methods and methodologies employed, and we removed duplicates, to create a final data set of 71 papers: 31 primary research publications, 22 evidence reviews, 14 theoretical and conceptual reviews, and 4 policy documents. We identified different perspectives of the story lines; GH drafted narrative summaries that, agreed by all authors, informed a final taxonomy of the literature. We agreed on two perspectives on ways of knowing integrated care (patient perspectives and conceptual models) and two perspectives on the story line of strategies (integrated care as an outcome of organizational strategies, or as the subject of critical and theoretical analysis).

## Findings

Table 1 presents our taxonomy of the literature on integrated care, encompassing four perspectives of integrated care: patients’ perspectives; organizational strategies and policies; conceptual models; and theoretical and critical analysis. We review these in turn.

**Table 1:** A Taxonomy of Integrated Care Literature

Key Perspectives	Main Focus of Papers	Lines of Argument^a^
**Patients’ Perspectives of Integrated Care (9 papers)**		
Redding, 2013; Reinhard, 2013	Eliciting patient's perspectives through consultation and focus groups.	Patients value person‐centered coordinated care, being able to control and plan their care, communication, continuity of care, and practical and emotional support.
Gowing et al., 2016; Greenfield et al., 2014; Hudon et al., 2015; Sargent et al., 2007; Spoorenberg et al., 2015	Subjective experiences of integrated care, including patients’ perspectives of integrated care programs, multidisciplinary case management, and satisfaction with care provided.	Reassurance and psychosocial support are important to patients and carers (but are not necessarily included in official guidance). For care to be person‐centered, patients need to be considered as active subjects.
Singer et al., 2011; Vrijhoef et al., 2009	How to conceptualize and measure integrated care from the patient perspective.	The object of integration should be patient care (as opposed to organizational integration), with patient centeredness as a key element. Measures of patients’ experiences and satisfaction of services can be undertaken through validated survey instruments.
**Organizational Strategies and Policies to Integrate Care (46 papers)**		
*Case Management (12 papers)*		
Baker, Grant, and Gopalan, 2018; Huntley et al., 2013; Reilly, Hughes, and Challis, 2010; Stokes et al., 2015	Evidence reviews: systematic reviews and meta‐analyses of the effectiveness of case management for multimorbidity and high service utilization, hospital admissions for older people, and “at‐risk” patients in primary care, and literature review of the implementation and processes of case management.	There is little evidence of effectiveness of case management on health care utilization or in reducing hospital admissions, and some evidence of improvement in patient‐reported outcomes.
Boaden et al., 2005; Carrier, 2012; Gravelle et al., 2006; Kane et al., 2003; Sheaff et al., 2009	Evaluation and research of case management, including the Evercare model in the United States and the United Kingdom;practice of case management in Canada and the United Kingdom.	The Evercare model of case management found to prevent hospitalizations and be cost‐effective in the United States was piloted in the United Kingdom but did not have the same effects. Case management practices were shaped by context of home care, size of caseloads, and availability of resources; although valued by patients and carers, little system change resulted from case management practices.
UK Department of Health, 2005; Ross, Curry, and Goodwin, 2011; NHS England, 2016	Policy and guidance on case management in the English NHS.	Case management needs to be targeted and proactive to be cost‐effective, being most effective when implemented as part of a wider program of integrated care.
*Multidisciplinary Working (8 papers)*		
Harris et al., 2012; Harris et al., 2013; Janse et al., 2016a; Janse et al., 2016b; Kassianos et al., 2015; Lusardi and Tomelleri, 2017; Raine et al., 2014; Tousijn and Willen, 2012	Primary research into the processes and effects of multidisciplinary team/group working, including extent and intensity of integration in multidisciplinary groups, experiences and perspectives of professionals, team relationships, and effect of multidisciplinary teams on implementation of treatment plans.	Multidisciplinary working is central to interventions to integrated care, with evidence of effectiveness in integrating care but with limited evidence of measurable effects of such integration on patients or system outcomes. Multidisciplinary working increases workload for professionals in terms of non‐patient‐related care, can have beneficial effects for professionals, has some effects on interprofessional relationships, and does not necessarily equate to more collaborative decisions or actions being implemented.
*Linking Organizations (14 papers)*		
*Coordination Technologies*		
Ahgren and Axelsson, 2011; Allen, Gillen, and Rixson, 2009; Haland and Rosstad, 2015	Policy analysis of chains of care in Sweden; systematic review of effectiveness of integrated care pathways (who they are effective for and in what circumstances); qualitative research of care pathways integrating primary and secondary care.	Care pathways and chains of care coordinate activities for patients/groups of patients across organizational boundaries, comply with best clinical practice, and distribute work required to support patients/groups of patients with complex needs.
*Partnership Working*		
Barker, 2014; Glasby, Dickinson, and Miller, 2011; Shaw and Rosen, 2013	Policy analysis of health and social care funding and partnership working in English settings and policy analysis of fragmentation.	Partnership working enables organizations to strategically address common concerns requiring multiagency solutions.
Macadam, 2015; Rudkjobing et al, 2014	Examples of interorganizational coordination in Canada (networked governance model) and Denmark (health care agreements).	Partnership working facilitates joint working between organizations through agreements and governance arrangements without merging or otherwise changing organizational structures.
*Collective Accountability*		
Hwang et al., 2013; Ramsay, Fulop, and Edwards, 2009	Reviews of evidence of effects of Integrated Delivery Systems on cost and quality evidence base for vertical integration in health care.	Integrated delivery systems have been introduced in the US setting to address concerns of fragmentation, cost, and variation in quality of care. Vertical integration can enable capture of cost savings that are related to providing upstream/preventive care.
McCarthy et al., 2009; Ovretveit, Hansson, and Brommels, 2010	Case studies of Kaiser Permanente and Norrtalje, Sweden.	Kaiser Permanente is an example of a successful integrated delivery system in terms of competitiveness in the health care market, and providing high quality for low cost and low use of hospital beds. Norrtaljie is an example of an integrated public health and social system influenced by a range of organizational and contextual factors.
Farmanova, Baker, and Cohen, 2019; King's Fund, 2018	Scoping review of strategies to develop integrated and population‐health‐focused health systems and policy review of accountable care proposals for England.	Integration of services can be combined with population health approaches; accountable care organizations and integrated delivery systems are forms of integration that can improve health outcomes at the population level.
*Whole System Approaches (12 papers)*		
Baxter et al., 2018; Beland and Hollander, 2011; Johri, Beland, and Bergman, 2003; Ouwens et al., 2005; Martinez‐Gonzalez et al., 2014	Evidence reviews (systematic review, evidence synthesis, review of systematic reviews and metareview) of effects of models of integrated care for frail, elderly, and adults with chronic conditions.	Strategies to integrate care for patients need to be supported by organizational, workforce, financial, and systems changes in a programmatic or whole system approach. Methodological problems arise in evaluating and comparing multifaceted/whole system programs in diverse settings.
Davy et al., 2015; Wagner, Austin, and Von Korff, 1996; Wagner et al., 2001	Development of the Chronic Care Model, a heuristic model for organizing care for chronically ill patients; application and systematic review of its effectiveness.	The mismatch between the kind of support people with chronic conditions need and that which is available from health systems can be addressed by effective system changes, summarized in the evidence‐based Chronic Care Model.
Nolte and McKee, 2008; Armitage et al., 2009; Ham, 2010; Suter et al., 2009	Analysis of health systems’ responses to caring for people with chronic conditions, effectiveness and impact of health systems integration, and characteristics of (a) high‐performing chronic care systems and (b) successfully integrated health systems.	A systems perspective provides insight into (a) the features of systems that create fragmentation of care and shortfalls in appropriate responses to people with chronic conditions and (b) the actions required to integrate systems and hence to generate improved service delivery and population health.
**Conceptual Models of Integrated Care (7 papers)**		
Fulop, Mowlem, and Edwards, 2005; Kodner and Kryiacou, 2000; Leutz, 1999; Singer et al., 2018; Valentijn et al., 2013	Synthesis of empirical work to define integrated care, extend conceptual understanding, and develop conceptual frameworks and explanatory models.	Comprehensive conceptual models can account for different typologies and degrees of integration, the components of integration, and the relationship between these components.
Kirst et al., 2017; Sheaff et al., 2018	Realist reviews/synthesis of processes that are associated with success of integrated care programs; evidence and assumptions about how new models of integrated care can change use of health care.	Realist approaches build theory about connections between outcomes, mechanisms, and context in successful integrated care programs.
**Theoretical and Critical Analysis (9 papers)**		
*Patient Trajectories*		
Allen, Griffiths, and Lyne, 2004; Nugus et al., 2010	Empirical studies from the United Kingdom and Australia of patients’ trajectories through complex service provision, drawing on theories of illness trajectories, game theory, and complex adaptive systems.	Patients’ trajectories are unpredictable, not random, and emerge from complex systems.
*Recursive Nature of Structure/Agency*		
Embuldeniya et al, 2018; Williams and Sullivan, 2009	Empirical study of integrated payment mechanisms in Canada, and integrated health and social care in Wales, drawing on practice theory, Bourdieu, and habitus.	Integration was iteratively generated by the recursive interplay between structures of integrated care and individuals’ actions.
*Social Organization of Work*		
Allen, 2014; Shaw et al., 2017	Empirical studies from the United Kingdom of the organization of work through integrated care pathways and patients’ transitions from hospital, drawing on boundary object theory and institutional logics.	The organization of work across and within organizations and institutions can be analyzed as a series of social processes or practices.
*Critical Analysis*		
Dickinson et al., 2013; Hammond et al., 2017; Pickard, 2009	With a focus on English health policy, critically analyzing joint commissioning, and analyzing discourses of (1) place and (2) old age.	Integrated care strategies perform other work in addition to their ostensive aims, shaped by political and social contexts and power relations.

a How integrated care is understood to manifest and affect change.

### Key Perspective 1: Patients’ Perspectives of Integrated Care

Integrated care programs sought to change *how* care is provided (changing the experience of care) rather than necessarily changing *what* care is provided. Understanding how, and to what extent, patients’ experiences of care are affected by integrated care programs was, therefore, of central importance to measuring the effectiveness of integrated care programs and, on a more philosophical level, understanding what integrated care is and what it means for patients. Associations between improved patient experiences and improved outcomes (see, for instance, the review of integrated care programs by Ouwens et al.^56^) appeared to be related to improved adherence to treatment guidelines,^23^, ^57^ improved access to care,^24^ and patient‐centered care (ie, “activated” patients have better health outcomes such as improved diabetic control).^58^ How patients experienced integrated care and what it meant to them was less clearly associated with organizational strategies and policies.

We included nine papers (see Table 1) that contributed to knowledge of integrated care by incorporating different patients’ perspectives. These papers asked what patients wanted from integrated care,^59^, ^60^ what person‐centered care meant for patients,^61^, ^62^ how patients experienced integrated care,^63^, ^64^, ^65^ and how patients’ perspectives can measure integrated care.^16^, ^53^

Two papers reported on consultative methods (focus groups, development and testing of statements) to engage patients and representative organizations in policy debates about what patients wanted from integrated care.^59^, ^60^ National Voices, a UK coalition of health and social care charities, was commissioned by the English NHS to define integrated care from a patient (or in their terminology, a service user) perspective. The resulting definition was “person‐centered, coordinated care,” meaning patients taking appropriate control of their own care: “I can plan my care with people who work together to understand me and my carer(s), allow me control, and bring together services to achieve the outcomes important to me.”^59^ ^(p332)^ Coordination of care, access to services, emotional support—“someone to look out for them”^60(p69)^—and practical support were important to the “dual eligibles” (ie, people who qualified for both Medicare and Medicaid, mainly older adults and people with disabilities) who contributed to focus groups in the United States.^60^

Interpretations of what person‐centered, coordinated care meant to patients were provided by qualitative studies conducted within wider evaluations of integrated care programs in the English NHS and the Netherlands^61^, ^62^ to examine patients’ narratives of person‐centered care and the extent to which it met their needs. Greenfield et al. characterized patients’ experiences of person‐centeredness as a sense of space to be seen and heard: “to be ‘seen’ as a whole person with a whole life beside their medical symptoms, and having psychological as well as medical needs.”^61^ ^(p5)^ Such person‐centeredness was not always achieved, leading authors to conclude that patients can be considered as passive objects of care, rather than active subjects. Spoorenberg et al. extended this line of inquiry by asking about patients’ experiences of care within the context of experiences of aging,^62^ finding that patients’ concerns about the consequences of aging (including fears of increasing dependency and poor health) related to and moderated their experiences of care. These studies extended ideas of person‐centered care by acknowledging patients’ active personhood and their contextualizing experiences.

High levels of patient and carer satisfaction with integrated care have been found, for example, in qualitative interview studies evaluating case management interventions in the English NHS^63^ and Canada.^65^ However, these studies also report that patients had little awareness or understanding of integrated care. Patients interviewed by Gowing et al. to evaluate an integrated care program expressed satisfaction with the health care professionals they had encountered, but were unaware of the program and its aims.^63^ This discrepancy between patients’ understanding of integrated care and that of service providers and policymakers reflects differences in what was valued. Qualitative research of case management in the English NHS found what was valued most by patients and carers—psychosocial support—was not included in policy guidance, leading the authors to describe psychosocial support as “implementation surplus” compared to the prescribed model of care.^64^ In short, there are differences between what patients understand and value and what is considered important from organizational and policy perspectives.

Methodological papers included in this review were concerned with patients’ perspectives as objective assessments—for example, surveys to ascertain the extent to which care is experienced as integrated by patients^16^ or to measure if components of the CCM (such as patient activation) are present and detectable by patients.^53^ Such methods of measuring integrated care, while concerned to establish if integrated care has an effect on patients, invariably impose a definition of integrated care that, given findings reviewed previously, will not necessarily accord with patients’ understanding of care.

In sum, we have found this literature to consider patients’ perspectives as formative (setting out that integrated care should be person‐centered and coordinated), interpretive (explicating meanings of person‐centered care), subjective (what it is like to experience integrated care), and objective (measuring if integrated care is present). Findings about what patients value in relation to integrated care (eg, psychosocial, emotional and practical support) and how they understand the care that is provided to them indicates that patients do not necessarily know or value the same concepts of integrated care (eg, case management) as health care providers and policymakers.

### Key Perspective 2: Strategies to Integrate Care

We included 46 papers examining organizational strategies and policies to integrate care (see Table 1). We categorized these into four related but analytically distinct strategies according to how they were expected to effect change: case management, multidisciplinary working, linking of organizations, and programmatic or “whole system” approaches.

#### Case Management

Case management is the predominant approach to integrating care for people with multimorbidities.^66^, ^67^ We included 12 papers on case management: four evidence reviews of effectiveness of case management for people with multimorbidities and high service utilization,^68^ effectiveness in reducing unplanned hospital admissions for older people,^55^ and for “at‐risk” patients in primary care,^69^ and the implementation and processes of case management^70^; five empirical papers, including a quasi‐experimental study of the Evercare model in the United States,^71^ two reports of the mixed‐method evaluation of Evercare in the English NHS,^8^, ^72^ and qualitative studies of case management in Canadian^73^ and English settings^74^; and three policy and practice guidance documents.^75^, ^76^, ^77^

Case management proved to be a “micro” example of integrated care, focusing on individual clinical management of patients, but one that had been deployed to achieve “macro,” or system‐level, goals, such as reducing hospital admissions. We found that case management focused on managing an individual patient (typically someone with complex needs or multimorbidity) through a set of activities undertaken by a professional (often a community‐based nurse) concerned with providing proactive, planned, preventive care and supporting patients with self‐management. However, case management has been widely evaluated in terms of effectiveness in reducing hospital admissions.

Systematic reviews of trials of case management in Australia, Canada, Denmark, Germany, Hong Kong, Italy, Sweden, the United States, and the United Kingdom have not demonstrated measurable reductions in hospital admissions.^55^, ^68^ The translation of the Evercare model of case management from the United States to the English NHS demonstrates the challenges of applying an intervention across different health care systems. The Evercare program, operated by United Health Care, a for‐profit variation of a US health maintenance organization, provided intensive case management in primary care to nursing home residents.^71^ Successful in reducing hospitalizations and improving cost effectiveness in the United States, Evercare failed to reduce hospital admissions in the English NHS.^72^ The failure was attributed to poor targeting; instead of case management being offered to patients who were most at risk of hospital admission, other patients not at risk of admission were accessing the service.^8^ The importance of accurately targeting patients for case management to have an effect on hospital admissions was further highlighted by analysis of routine admissions data in the English NHS, which found that patients who had been admitted were likely to have fewer admissions without any intervention.^78^ Consequently, predictive risk modeling (risk stratification for case finding) has been routinely incorporated into integrated care programs to identify people who are considered to be at risk of future hospital admissions and target those people for case management.

Analysis of wider structural and organizational factors helps to explain why case management might not work to reduce hospital admissions. Purdy and colleagues found structural factors amongst those factors associated with risks for avoidable hospital admissions: age and deprivation, ethnicity, distance to hospital, rurality, lifestyle, meteorological factors, and pollution (the latter have consequences for people with respiratory problems).^79^ Such factors are unlikely to be modifiable through case management. Organizational factors, such as high caseloads for case management and limited resources with which to respond, were identified by Carrier as constraints on the potential for case management to be proactive and preventive in terms of hospital admissions.^73^ Sheaff and colleagues found that case management did not lead to any of the reengineering of services that the authors indicated would be needed to change the rate of hospital admissions.^74^

In sum, case management has been targeted at patients identified to be at most risk of future hospital admission to improve cost effectiveness and reduce hospitalization. Studies of the effectiveness and practice of case management indicate that a microlevel strategy focused on coordinating care for individual patients is unlikely to achieve system‐level outcomes.

#### Multidisciplinary Working

Multidisciplinary working entails health and social care professionals working collaboratively across different settings to manage specific patients. Arguably the single most important mechanism for care coordination,^32^ multidisciplinary working has been a central focus of many integrated care programs for people with chronic conditions and multimorbidity^11^, ^66^, ^67^, ^80^, ^81^ and has commonly been associated with case management. Case managers have often worked within multidisciplinary teams, crossing disciplinary boundaries to coordinate care across multiple services. We included eight primary studies of multidisciplinary working in community settings with patients with chronic illnesses: two that analyzed the extent and intensity of integration in multidisciplinary groups using content analysis of talk in meetings,^82^, ^83^ a mixed‐methods study of the effectiveness of multidisciplinary teams in terms of implementation of treatment plans,^80^ an ethnographic study of how integration was produced in multidisciplinary teams,^84^ two interview studies examining perspectives on interprofessional relations^85^ and processes of implementation,^86^ and two quasi‐experimental studies examining the impact on professionals^87^ and on processes of integration.^88^

We found that multidisciplinary working was concerned with the clinical integration of care for individual patients and with cross‐professional or transdisciplinary working (with professionals expected to “transcend” their own field^82^). The effects of multidisciplinary working were anticipated to be felt beyond specific patients, as collaborative working was expected to aid improvements in productivity and efficiency as a result of better coordination and networking.^83^

Studies of professionals’ views indicated that multidisciplinary working takes considerable time and effort. Professionals in the Netherlands experienced a greater burden of work and spent more time on non‐patient‐related tasks (eg, meetings, referrals, administration) than those providing usual care. Despite additional burdens, experiences of multidisciplinary working were not reported as reducing job satisfaction.^87^ On the contrary, opportunities for cross‐sector learning and developing holistic approaches were welcomed by professionals in the English NHS as positive effects of multidisciplinary working, although they also identified some barriers to effective working in their multidisciplinary groups.^86^

Multidisciplinary working changed relations between professionals, but not to the extent of effecting changes for patients or the wider system.^82^ Janse and colleagues found that collaborative relationships between professionals were facilitated by organizational changes such as multidisciplinary team meetings and shared protocols.^88^ However, multidisciplinary working did not alter professional roles and hierarchies. In their study of interprofessional relations, Tousijn and Willem found that traditional patterns of medical dominance in health and social care teams persisted despite the development of participatory team dynamics.^85^ Similar patterns were found by Harris and colleagues.^82^ Even when cultural integration (defined by Lusardi and Tomelleri as shared knowledge, values, and goals) was fostered through collaborative working, health and social care professionals continued with their differing professional practices.^84^

Multidisciplinary working is considered central to integrated care strategies. While patients’ views on multidisciplinary working have been found to be broadly positive, there is little evidence of significant changes in interprofessional relationships and subsequent benefits for patients and systems.

#### Linking Organizations

Interorganizational integration is a common feature of integrated care strategies to coordinate care across services. Where multiple teams, services, or organizations are involved in supporting people with complex needs, they need to be appropriately linked to provide the continuum of care required. We identified 14 papers concerned with organizational integration and from these found three types of linkages between organizations: coordination technologies (integrated care pathways and chains of care); partnership working (including collaborative agreements); and collective accountability (financial and clinical). We consider these in turn.

##### Coordination Technologies

Coordination technologies apply evidence‐based guidelines to the organization of care for specific conditions in order to improve quality and safety. We included three papers on coordination technologies (see Table 1): a systematic review of the effectiveness of integrated care pathways,^89^ qualitative research of a care pathway to integrate primary and secondary care,^90^ and a historical account and policy analysis of “chains of care.”^6^

We found that care pathways and chains of care, despite different conceptual underpinnings, were both expected to establish efficient and effective processes across services funded or provided by multiple organizations. Coordination technologies specified the clinical care required by individual patients and supported planning and purchasing (commissioning) of care for groups of patients. A systematic review of the effectiveness of integrated care pathways found they mapped patients’ journeys to guide the provision of care according to best clinical practice and in line with clinical governance processes. Integrated care pathways improved adherence to evidence‐based practice (they worked best when patients’ needs were predictable) and formalized the input of multidisciplinary teams.^89^ Primary research into a care pathway in practice showed how it enabled renegotiation of the boundary between hospital and home care for older people requiring home care after hospital discharge.^90^

Chains of care was developed in Sweden in the early 2000s to improve interorganizational integration and manage the increasing complexity of health care. Chains of care were contractual agreements between organizations (based on clinical guidance) specifying how services should be provided for certain groups of patients. Like integrated care pathways, chains of care are intended to coordinate activities for patients across multiple providers in order to improve quality of care, taking a similar linear approach to conceiving how care might be organized over time. However, unlike integrated care pathways, they have an organizational rather than individual patient focus and are informed by manufacturing processes (eg, organizing activities as sequential, repetitive processes with predetermined outcomes) in addition to clinical evidence. The resulting differentiation of tasks and allocation to teams or organizations requires integration to achieve “unity of effort.”^91^ ^(p76)^

In sum, integrated care pathways and chains of care stem from different conceptual frameworks but aim to distribute clinical work appropriately across the multiple organizations or services involved in providing care to patients with chronic and complex needs.

##### Partnership Working

We included five papers on partnership working (see Table 1): an analysis of the policy problem of fragmentation that integrated care seeks to address,^92^ two policy analyses of health and social care partnerships in England,^93^, ^94^ a case study of a networked governance model in Canada,^95^ and a survey study of health care agreements in Denmark.^96^ The primary concern of all five papers was with linking organizations through strategic alliances and shared governance arrangements.

We found that partnership working strategically and practically linked organizations to address common policy problems (including fragmentation of care) that required multiagency solutions and cooperation. Take the example of long‐term care for older people in England, where the historic separation of health and social care has divided responsibilities as well as allocation of costs for residential, nursing, and hospital care. The provision and funding of such care has typically involved a complex set of contributions from central government, local government, NHS, private provision, and individual funding. Different funding rules—health care being free at the point of access and social care being means‐tested—have been found to cause confusion and inequities^93^ and prove challenging for care coordination. In England, policy solutions to these problems have included strategies to integrate health and social care through joint governance arrangements (including joint commissioning,^97^ joint health and well‐being boards,^98^ and devolution initiatives^99^) that seek to tackle complex problems collaboratively.

We found that, in settings where acute and long‐term care were organized and funded by separate government agencies or levels of government, integrated governance structures were established to coordinate responsibilities and costs—for example, health care agreements to coordinate health and social care across administrative levels in Denmark.^96^ In Canada, the Program of Research to Integrate Services for the Maintenance of Autonomy (PRISMA) model of care established a consistent model of coordinated care for elderly people with chronic conditions across multiple agencies through shared governance structures (management committees) to oversee consistency of service delivery while allowing participating agencies to maintain their organizational independence.^95^

In sum, this literature highlights how partnership working facilitates multiagency cooperation in tackling complex problems, through shared intentions and agreements, without altering organizational boundaries.

##### Collective Accountability

Collective accountability across organizations can be achieved by integrating separate organizations into a unified whole, either through organizational merger into a single financial entity or through creation of an integrated delivery system held to account via contractual arrangements. We included six papers focused on unified organizations or delivery systems (see Table 1): three evidence reviews of the effects of integrated delivery systems,^25^ vertical integration,^100^ and strategies to develop integrated and population‐health‐focused health systems^101^; two empirical case studies of integrated delivery systems from Sweden^102^ and the United States^103^; and a policy briefing on accountable care proposals for the English NHS.^104^

All six papers highlighted a common concern with getting “best value health care” (low cost and high benefit), oriented to preventive, proactive, coordinated primary care that minimizes acute and hospital care and leads to better patient outcomes and lower costs. Integrated payment mechanisms were intended to incentivize high‐value care. Capitated or bundled payments (eg, as introduced in Canadian integrated funding models^1^) are offered to health care providers (or groups of providers) as a predetermined fee in return for delivering agreed outcomes for defined populations (such as residents of a specific geographic area or for an enrolled group of patients) for a specific time period. This approach shifts away from funding on a fee‐for‐service basis that is not only associated with fragmented care but also has been found to exclude fees for coordination activities.^105^ High‐value (preventive or “upstream”) care is therefore incentivized as providers seek to minimize costs in order to remain within their financial envelope, which is set either by insurance premiums or by government allocations according to the health system context.

Vertical integration has been stimulated by these changes to payment mechanisms. Ramsay, Fulop, and Edwards defined vertical integration as either a single organization taking control of the whole care pathway (ie, supply chain) or integration between provider and purchaser to allow the purchaser to share the financial benefits of preventive care.^100^ Kaiser Permanente provided an example of a single delivery system incorporating the health plan (ie, the purchaser of health care) with providers of health care.^103^ Regarded as an exemplar of integrated care, Kaiser Permanente has performed well in terms of finances, quality of care, and low use of hospital beds, indicating high‐value care.^106^ These attributes were associated with the integrated nature of the organization, which allowed it to develop population‐based strategies, stratifying all enrollees of the system to receive levels targeted to their needs.

The Swedish networked delivery system of Norrtajle provided another example of a single organization purchasing, providing, and governing health and social care for a defined population, in this case all residents of the region.^102^ In English health policy, a similar “place‐based” approach to developing integrated delivery systems has been pursued to encompass a population health approach.^104^ The expectation is that these systems will be financially accountable for providing care for their resident population within allocated funds, and clinically accountable for achieving national standards and targets.

Developing collective accountability appeared to align the interests of individual patients, purchasers, providers, and populations around low‐cost, high‐value care (or the “triple aim”^107^). Moreover, the benefits of a single organization providing a breadth of care, offering the full range of services needed by people and populations with complex needs was considered to offer the optimum way of improving care continuity, coordination, and patient experience. See, for example, Sheaff's commentary, informed by a realist synthesis of evidence of multispecialty community providers.^108^

In sum, technologies, partnerships, and collective accountability offer different strategies to integrate care by creating different kinds of linkages between organizations. Coordination technologies utilize technical resources to distribute care effectively across multiple providers; partnership working enables organizations to address shared concerns; and creating collective accountability incentivizes high‐value care for defined populations, stimulating the creation of integrated organizations or delivery systems.

#### Whole System Approaches

Evidence of strategies to integrate patient care, develop multidisciplinary working, and link organizations suggests that any of these methods pursued in isolation will not be sufficient to make meaningful, measurable changes. Instead, a multifaceted, or whole system, approach similar to those taken by demonstration projects or programs introducing entire new models of care might be required. Whole system models seek to implement multiple, interrelated changes to how services are organized; the CCM also sought to change aspects of the system within which the model is being introduced. An alternative interpretation of a “system” perspective is to analyze the extent to which the health system within which the integrated care model is being introduced is integrated or supports integration. We included 12 papers with a systems approach (see Table 1): five evidence reviews of multifaceted experiments and models of integrated care for frail elderly people and people with chronic conditions^4^, ^23^, ^24^, ^30^, ^56^; three that developed, assessed, and systematically reviewed the application of the CCM^57^, ^109^, ^110^; and four that analyzed the extent to which health systems were integrated.^2^, ^39^, ^111^, ^112^

Demonstration programs, experiments, and new models of integrated care commonly aim to address multiple issues simultaneously, such as patient care, workforce interventions, and organizational, financial, and system changes.^24^ An early evidence synthesis of integrated care demonstration programs—for example, the Program of All Inclusive Care for the Elderly, which provided targeted, comprehensive care integrated via a day center and case management—explains how they combined financial and organizational changes with clinical and managerial techniques to create new kinds of delivery systems.^4^ The study authors concluded that a comprehensive approach to restructuring services was most likely to succeed in terms of reducing costs and improving care. Subsequent systematic reviews have found that whereas integrated care programs have had similar aims of reducing fragmentation and improving continuity and coordination, their content and focus have differed,^23^, ^56^ although core components, including multidisciplinary care and case management, have been commonly found in successful programs.

The CCM proposed changes to the wider context within which services are located. The CCM synthesized evidence of changes needed to facilitate high‐quality chronic disease care into a heuristic model that connects clinical practice, community resources, and health system. Based on addressing the common challenges that chronic patients experience in relation to their conditions, including symptoms, challenges of managing treatment, and emotional impact, the CCM aimed to support patients to develop skills and confidence in taking active and informed roles in their care and to create the optimum conditions for the provision of chronic care.^109^ The CCM has widely influenced health care improvement programs in the United States,^110^, ^113^ the United Kingdom,^75^ and other high‐income countries^57^ and has provided a conceptual framework to guide and evaluate integrated care systems and programs,^39^, ^114^ as well as informing the Patient Centered Medical Home, a new model of primary care.^40^

An alternative systems perspective involves understanding the extent to which an existing national or regional health system is integrated. Nolte and McKee presented a comparative analysis of contextual system factors to assess how the overall national policy environment impinges on the provision of chronic care and integrated care.^112^ Other scholars have examined the extent to which systems are integrated^111^ and the components and characteristics of successfully integrated^2^ and high‐performing chronic care^39^ systems. By taking this system perspective when analyzing integrated care programs, researchers have found that wider issues such as universal coverage and health care free at the point of access^39^ become relevant. Similarly important at a system level is the provision of high‐quality primary care, which is associated with improved outcomes. In many health systems, primary care practitioners undertake coordinating and gatekeeping roles.^115^ A health systems perspective explains why certain strategies are more important in certain settings, with the US focus on defined populations and integration of financing and provision of care compared to the European focus on integrating the “cure and care” sectors. We conclude that whole system perspectives result in development and implementation of models that comprise multiple components and recognize system characteristics such as universal primary care as prerequisites for integrated care.

Our review of the literature on the strategies and policies to integrate care found multiple objects of integration: care for individuals and groups of patients; multidisciplinary teams; complex policy problems; financial, clinical, and managerial accountability; and whole system approaches. These objects were pursued for a range of reasons—to improve care for individuals but also to contribute to system goals of efficiency and population health outcomes.

### Key Perspective 3: Conceptual Models of Integrated Care

Scholarly work to define, measure, and compare integrated care programs has resulted in conceptual frameworks that are intended to support the implementation and comparative measurement of integrated care in different contexts. We included seven papers (see Table 1) that sought to conceptualize integrated care: five setting out theoretical models of integrated care^28^, ^29^, ^36^, ^37^, ^116^ and two using realist approaches to develop theories of how integration leads to improved outcomes.^31^, ^32^

The concept of integration as a unified ideal was first proposed by Kodner and Kyriacou^29^ and Leutz^28^ as a spectrum, running from linkages (referrals between services) through coordination (smoothing transitions between services) to full integration (into a single organizational body), with a range of factors affecting the extent to which integration might be achieved. Fulop, Mowlem, and Edwards's review of empirical examples resulted in a more complex framework that introduced four levels—organizational, functional, service, and clinical—at which integration could take place and two types of integration associated with success—normative integration or shared values, and systemic integration, or coherence of rules and policies.^36^ This was a unified model, with each of the levels and types of integration required to achieve effective integration.

Valentijn and colleagues extended Fulop's model to incorporate the integrative functions of primary care into a multilevel model that included a population health perspective.^37^ The resulting “rainbow” model had six dimensions of integrated care (clinical, professional, organizational, system, functional, and normative) thought to operate at three levels (micro, meso, and macro), connected by normative (social norms, mutually shared goals) and functional integration (coordination of support services such as finance, management, and informatics) to simultaneously deliver person‐focused, population‐based care. Singer and colleagues’ comprehensive theory of integration^116^ extended this conceptual work inthree ways: by synthesizing and extending the dimensions of the models (in particular, elaborating the distinction between normative integration as being a specific culture of coordination, and interpersonal integration as comprising collaboration and teamwork); hypothesizing relationships between components of the model; and identifying aspects that can be more or less easily influenced. The resulting model is more dynamic in that it accounts for potential conflict within the model between the interests of different stakeholders, and less deterministic in that it recognizes different kinds of relationships that exist within the model and between the model and the wider context.

In contrast to unified models of integrated care offering comprehensive theoretical explanations for how integration can be achieved, realist reviews build program theories from empirical evidence as to how change might be expected to happen by considering the interplay among context, mechanisms, and outcomes. Kirst and colleagues’ realist review elucidated the connections between programs (changes such as establishing multidisciplinary teams), context (including strong leadership and organizational culture), and the desired outcomes (reduced service utilization, improved outcomes and experiences).^31^ Sheaff and colleagues took a similar approach to synthesize evidence and test policy assumptions about how models of care might shift service use from hospital to enhanced primary care.^32^

Realist approaches, which synthesize processes of change, emphasize the importance of context for integrated care programs, whereas unified conceptual models abstract the components of integrated care from their context. Unified models create normative approaches toward integrated care as redistribution of care activities, changes in financial incentives, and new organizational forms are associated with successful integration. However, when the issue of context is reintroduced as a component of unified models, such as in the CCM, it becomes another factor to be incorporated into a program of change. Efforts to implement models of integrated care become, in the breadth of their ambition, analogous with efforts to create ideal health systems.

### Key Perspective 4: Theoretical and Critical Analysis

We included nine papers that brought social theories to the analysis of empirical studies of integrated care (see Table 1). They did this by developing concepts of patient trajectories,^117^, ^118^ examining structure and agency,^1^, ^119^ analyzing the social processes of work,^120^, ^121^ and critically analyzing power relations inherent in integrated care strategies.^122^, ^123^, ^124^ We consider these in turn.

Patients cross organizational and disciplinary boundaries as they move along care pathways or as they seek help from multiple services for different needs. Allen, Griffiths, and Lyne used the concept of *care trajectories* to analyze how multiple services supported a patient's rehabilitation.^117^ The authors theorized that patients’ trajectories were unpredictable, yet not random. Patients’ experiences as they accessed multiple health and care services could be understood as sequences of interactions with norms or rules that are generally followed (similar to the rules of a game). The choices patients made were shaped by the ways in which services were organized. Nugus and colleagues extended the concept of patient trajectories in their study of the dynamic processes of integration in emergency hospital care.^118^ Adopting a complex adaptive system perspective—understanding the health care system as made up of dynamic, interdependent relationships and activities, open to influence and change from the wider environment—they found that patient trajectories, often understood as linear from a continuity of care perspective, could also be understood as complex, emerging from multiple, dynamic relationships.

Williams and Sullivan applied the sociological concepts of structure (structural factors included policy, national programs, resources) and agency (eg, the work of individual managers) to explain how integration was produced in a case study of integrated health and social care.^119^ Embuldeniya and colleagues took a realist approach to study how integration was generated from an interplay among context, mechanisms, and outcomes.^1^ The authors drew on Bourdieu's concept of habitus^120^ to analyze the iterative relationship between contexts and mechanisms, characterizing the coming together of integrated care program structures and systems as *connectivity* and the coming together of people and ideas as *consensus*. In this case, integration was generated iteratively by the recursive interplay between connectivity and consensus.

The organization of work across and within institutions was analyzed as a series of social processes by Allen in relation to integrated care pathways^121^ and by Shaw and colleagues in relation to transitions of patients from hospital into community services.^122^ Allen extended boundary object theory—boundary objects being material or conceptual objects that create collaborative space and contribute to changing relations between people—to analyze how integrated care pathways bridged divisions between services by reorganizing work. Shaw and colleagues analyzed the connections among the micro, meso, and macro processes of integrated care to find that macro institutional ideas (in this case, the legislation that endorsed partnership working between hospital and social services) influenced meso organizational logics, but that individuals’ actions were critical to changes at the micro level.^122^

We included critical analysis of three strategies to integrate care in the English NHS: case management, joint commissioning, and place‐based planning.^123^, ^124^, ^125^ A common finding was that such strategies did not simply work as neutral coordination devices, but could be analyzed as sites of political action. Pickard, for example, took a Foucauldian approach, focusing on discourses of knowledge and power to analyze case management.^125^ The author problematized the targeting of individuals for proactive case management as an attempt to “govern old age.” Identifying older people as “at risk” and hence requiring the intervention of case management had emerged from a particular understanding of old age as being a problem for society. Contextualizing case management within these particular historical and cultural discourses about risk and old age revealed structures of power and expertise that allowed case management to be practiced.

Critical analysis of joint commissioning and place‐based care in the English NHS found that these strategies had effects other than the primary aims of improving outcomes. An analysis of joint commissioning concluded that it was not a means to an end, given that there was little evidence of improved outcomes, but that it performed other kinds of work—depoliticizing decisions that enabled demand for services to be managed, and avoidance of issues of inequality.^123^ Hammond and colleagues analyzed the claims for accountable care as being able to improve population health and manage the financial pressures of health care.^124^ The implicit power relations in the development of place‐based care in the English NHS were interpreted as attempting to spatially and financially “fix” control over budgets for specified places. Implementation of accountable care was understood as a way of defining a financial envelope, allocating resources rather than accountability.

We found that these theoretically informed studies shifted from deterministic views of integrated care programs resulting in organizational changes and improved patient outcomes. Instead, this body of literature highlighted the recursive nature of structure and agency and the social practices involved in the organization of work as factors to consider in understanding how integrated care might be achieved and how it might benefit patients. Critical analysis of English strategies to integrate care showed how context shapes the problems integrated care is designed to address, such as old age and coordinating and planning care across multiple services; the solutions to those problems, such as joint commissioning and place‐based care; and the potential effects of integrated care programs beyond the intended outcomes of better coordinated patient care. Through this lens integrated care can be understood not only as a series of strategies to unify care and organizations but also as part of the political economy of health care, shaped by economic, political, and social contexts and subject to the governance and power relations that influence the means of funding and producing health care.

## Discussion

This hermeneutic review has extended analysis of the integrated care literature by summarizing a wide body of empirical evidence and theoretical work on integrated care. In doing so, we identified four framings of integrated care: patients’ perspectives, organizational strategies and policies, conceptual models, and theoretical and critical analysis. We identified important commonalities, tensions, and gaps across this body of work; common concerns to create unified frameworks; tensions between the idea of unity and the diverse practices of integrated care; and gaps between expectations for what integrated care should achieve and evidence of actual change.

The search for integrated care is a search for unity. This applies both to efforts to integrate patient care, services, and organizations and to scholarly work to create unified conceptual models. However, we found two key tensions across the literature that undermine the idea of integrated care as a unified concept. First, we found that “integrated care” was not one empirical phenomenon; rather, it covered a multiplicity of objects, strategies, and aims. Practical efforts to integrate care were concerned with creating unity of a great variety of objects: patient care and experience, multidisciplinary and interorganizational working, and health care systems. “Integrated care” included overlapping, interrelated, and, at times, conflicting strategies and experiences. Moreover, integration was pursued for a variety of ends (eg, to both improve patient experience and to reduce hospital admissions) and meant different things to different people. Patients, service providers, and policymakers had different ideas about, and different experiences of, integrated care.

Second, we found that conceptual models of integrated care assumed alignment of patient and system perspectives and of multiple strategies into a coherent, decontextualized whole. However, the range of framings provided by scholars bringing social theory and critical analysis to the study of integrated care undermines the normative, and often deterministic, frameworks provided by unified models.

Related to these tensions, we found gaps between organizational actions and outcomes for patients. Based on our review, we offer three linked explanations for these gaps. First, structural and social factors, such as allocation of resources and interpersonal dynamics, appear to constrain the ability of professionals to integrate—for example, through case management or multidisciplinary working. Second, patients are not necessarily able to fully exercise control over their care or to self‐manage. Their experiences are shaped not just by their own agency but also by the “rules of the game,” that is, the norms and structures of health care services and wider contexts of experiences, such as aging. Finally, even when integrated patient care is considered to be objectively achieved it might not be subjectively experienced as such by the patient if they are not, for example, provided with the space or time to be seen as a whole person. Patients’ subjective experiences are not determined by external factors such as services but produced by, among other factors, individual responses and choices.^117^, ^125^ When patients do experience and value person‐centered care, this is associated with relationship‐based care—psychosocial support, feeling cared for, and involved. These nonlinear connections between professional and organizational efforts on the one hand and patients’ experiences and outcomes on the other, undermine the assumption that improved patient care and experience can be determined by organizational and policy changes and support the case for a complexity‐informed approach to understanding the dynamics of integrated care programs and patient outcomes and experiences.

Despite these tensions and gaps, organizational and policy efforts to integrate care continue. This makes sense in light of our interpretation of the literature that these efforts are not just about achieving improved outcomes (or indeed about integrating care). They are also about how to organize the work of providing health and other related social and welfare services to people with complex needs across differentiated organizations and how to organize resources within different systems of funding and producing health care. Recognizing rather than resolving these tensions would mean accepting that relationship‐based care offering time and space for patients to be seen and heard is likely to be a necessary (if not sufficient) condition that can contribute to the achievement of integrated patient care. Moreover, provision of this care needs to be understood as not necessarily aligning with organizational or system objectives of reduced cost.

Comparative and critical interpretations illuminate how efforts to integrate care are part of the political economy of health care. Integrated care programs are not neutral but enact and reproduce power relations, as seen in critical analysis of English policies where integrated care strategies are implicated in organizational and political dynamics relating to resources and power. Normative models of integrated care assume that programs are pursued with an intention of achieving certain ends. However, the purposeful nature of programs that set out to improve systems and services needs to be considered in relation to the contexts that stimulate these changes. We have found from critical analysis that integrated care strategies emerge from particular historical, cultural, and social processes. Integrated care is a “common logic,” emerging from the particular “accidental” logics of different health care systems.^126^ As a policy response in particular settings, integrated care can also be understood as not simply a means to an end but as part of longer‐running debates about the allocation of resources, the role of the state, and the organization of health care and other public services.^127^ Interpretation of integrated care programs as emerging from complex social contexts belies the idea of integrated care acting as an intervention that can determine certain outcomes. Rather, integrated care comprises a set of social processes and practices that can contribute to, or detract from, the conditions that might enable improved outcomes or experiences.

Integrated care programs are contextually shaped but have common logics. Normative models of integrated care can therefore be of heuristic value (for example, in accounting for features of successful programs), but they will inherently fall short of accounting for the intricacies of different contexts and processes. The range of theoretical framings found in this review used to analyze integrated care preclude the creation of a single explanatory model. Integrated care can more usefully be studied and understood as comprising an emergent set of practices intrinsically shaped by contextual factors than as an intervention that will achieve a predetermined set of outcomes.

## Conclusion

In asking the question “what does it mean to integrate care?” we identified an array of strategies and conceptual work. The limitations of conceptual models in explaining equivocal and uncertain results are clarified by recourse to social theories that undermine deterministic models of integrated care. Critical analysis shows how the context of the political economy contributes to the pursuit of integrated care for a range of political and social reasons, notwithstanding the potential for benefits for patients. In light of these diverse concepts, strategies, and contextual factors, we caution against attempts to seek an ideal of unity of experience, practice, and theory and instead advise a rethinking of integrated care. We conclude that embracing ideas of complexity can open up opportunities for understanding integrated care as multiple, dynamic, emergent, and inseparable from context.

## Summary of Search Criteria

**Table:**

**Database**				
PubMed, Embase, Cumulative Index of Nursing and Allied Health Literature, Scopus, and Web of Science				
**Search Terms**				
OR		OR		OR
integrated care		aging		hospitalization
integrated system		elderly		hospitalisation
integrated delivery system		older people		hospital admissions
integration		frail		service utilization
community		long term conditions		service utilisation
community care	AND	chronic disease	AND	service costs
community setting		multi‐morbidity		emergency admissions
home		complex needs		non‐elective admissions
home care				
primary care				
general practice				
**Exclusions**				
Studies of children or young people under the age of 18, animal studies, papers not written in English, studies primarily of single conditions, those primarily concerned with injury (including brain injury) and serious mental illness (psychotic illnesses and personality disorder), studies of simple interventions, and studies where one intervention (for example, falls prevention, palliative care, behavioral health) was integrated into another.				
